# Life-time Actionable Pharmacogenetic Drug Use: A Population-based Cohort Study in 86 040 Young People With and Without Mental Disorders in Denmark

**DOI:** 10.1055/a-1655-9500

**Published:** 2021-11-09

**Authors:** Carin A.T.C. Lunenburg, Kazi Ishtiak-Ahmed, Thomas Werge, Christiane Gasse

**Affiliations:** 1 Department of Affective Disorders, Aarhus University Hospital Psychiatry, Aarhus, Denmark; 2 Department of Clinical Medicine, Aarhus University, Aarhus, Denmark; 3 Institute of Biological Psychiatry, Mental Health Services, Copenhagen University Hospital, Copenhagen, Denmark; 4 Department of Clinical Medicine, University of Copenhagen, Copenhagen, Denmark; 5 Lundbeck Foundation Center for GeoGenetics, GLOBE Institute, University of Copenhagen, Copenhagen, Denmark; 6 The Lundbeck Foundation Initiative for Integrative Psychiatric Research, iPSYCH, Copenhagen, Denmark; 7 Psychosis Research Unit, Aarhus University Hospital Psychiatry, Aarhus, Denmark; 8 Centre for Integrated Register-based Research Aarhus University (CIRRAU), Aarhus, Denmark

**Keywords:** Pharmacoepidemiology, psychopharmacology, genetics, testing

## Abstract

**Objective** To describe life-time use of current actionable pharmacogenetic (PGx) somatic and psychotropic drugs according to international PGx consortia in people with and without hospital-diagnosed mental disorders in the Danish population.

**Methods** Population- and register-based observational drug utilization study in 56 065 individuals with mental disorders, i. e. attention-deficit/hyperactivity disorder, autism, bipolar disorder, depression and schizophrenia, and a random, representative sample of 29 975 individuals of the Danish population, born between 1981 and 2005. Individuals were followed from 1995 or birth until 2016 (for a maximum of 22 years). We report prevalence and incidence rates of PGx drug use by age, sex and mental disorders based on redeemed prescriptions between 1995 and 2016.

**Results** Of the 69 PGx drugs, prescriptions of 39 drugs had been redeemed by the study population by 35 years of age. The use of at least 1 PGx drug varied between 23.1% in males without mental disorders and 97.2% in females with schizophrenia. Males with ADHD or autism were the youngest first-time PGx drug users at a mean of 11.6 years. The mean number of different PGx drugs used was 1.2 in males without mental disorders and 5.6 in individuals with schizophrenia. The prevalence of different PGx drugs linked to more than one gene was 25.3% in males without mental disorders to 94.1% in females with schizophrenia.

**Conclusion** PGx drugs are commonly used by younger people, more often by individuals with mental disorders and by females. Panel-based PGx testing could contribute to treatment decisions at a very young age.

## Introduction

Pharmacogenetics (PGx) is the study of the genetic predisposition of individuals, which can result in variability in drug metabolism, pharmacodynamics, or immunogenicity linked to treatment (non-)response or adverse events 1 . PGx aims at optimising treatment outcomes (i. e. increased efficacy or reduced risk of adverse events) by personalising treatment for patients based on their genetic makeup. Actionable PGx drugs are those for which drug or dosing recommendations have been made available by international PGx consortia in evidence-based PGx guidelines 2 3 4 . PGx guidelines have been provided for a proportionally higher number of psychotropic drugs than other drug classes 5 . Consequently, PGx testing could be of specific benefit for individuals with mental disorders, who often experience adverse events and delayed, insufficient, or non-response to psychopharmacological treatment 6 7 . Nevertheless, the implementation of PGx testing in psychiatry is lagging behind other specialities such as oncology 5 8 9 . The reasons for this have been discussed in recent review articles with a common ground of identified numerous barriers including perceived missing clinical evidence and utility, and knowledge and consensus about PGx testing among psychiatrists 5 9 .

Recently, several observational drug utilization studies of both somatic and psychotropic drugs with actionable PGx implications (PGx drugs) in different populations have contributed to a broader understanding of the potential impact of PGx testing based on the frequency of PGx drug use in larger populations 10 11 12 13 14 15 . The findings of these studies indicate, for example, that 50% of people using prescription drugs received one or more actionable PGx drug(s) during a 4-year period, 23% of first-time drug prescriptions included actionable drug-gene interactions (DGIs) and in approximately 25% of prescriptions, dose adjustments were recommended 11 12 13 14 . A preponderance of actionable psychotropic PGx drug use and the involvement of cytochrome-P450 (CYP) enzymes 2C19 and 2D6 DGIs were commonly observed 10 11 12 13 14 15 . Although people with mental disorders have not been the focus of these studies and thus, the incidence and prevalence of PGx drug use in people with mental disorders in comparison with an unselected population have not been investigated in detail yet.

One of the reasons for the low implementation of PGx guided dosing in psychiatry is a lack of consensus and specific advice regarding when to test to obtain the optimum and timely benefits. Several options regarding the timing of PGx testing are available, e. g. *i* ) the currently often used ˋreactive testingˊ when a patient experiences drug toxicity or non-response, *ii* ) ˋreactive prospective testingˊ by executing a PGx test at the time of prescribing to know the PGx status of the patient prior to the start of treatment, and *iii* ) ˋpre-emptive testingˊ by executing a panel-based PGx test at a certain time in a personˊs life, to know his or her PGx status prior to all future PGx drug prescriptions 11 . A panel-based PGx test including multiple variants in different genes has advantages over single-gene testing in that PGx guided dosing can be applied to PGx drugs with more than one established associated gene (i. e. actionable DGI), or when multiple PGx drugs are used subsequently with different DGIs or concomitantly resulting in drug-drug-gene interactions (DDGI). In addition, panel-based testing offers combinatorial PGx, in which multiple variants of different genes can be interpreted simultaneously to provide a more accurate personal PGx based dosing advice 16 17 . While panel-based testing has been advocated more recently 5 9 13 18 , guidance on whether to test pre-emptively or at which age first PGx drug use can be expected is still missing, which could be supported by observational studies investigating the exposure of actionable PGx drugs since birth.

## Aims of the study

To describe prescription drug use of current actionable somatic and psychotropic PGx drugs according to international PGx consortia in people with and without hospital diagnosed mental disorders in the Danish population. The specific aims were to investigate (1) the (life-time) incidence and prevalence of PGx drug use, (2) age at first PGx drug prescription, (3) the mean number of different PGx drugs per individual considering panel-based PGx testing (versus single-gene) and (4) the frequency of PGx drug use related to different genes regarding combinatorial PGx interpretation.

## Materials & Methods

### Study design

This was a population- and register-based cohort study of individuals born between 1981 and 2005 investigating prescription drug use of PGx drugs in Denmark between 1995 and 2016. The study used data of the Integrative Psychiatric Research (iPSYCH) consortium, which has established a large, unique Danish psychiatry-focused population-based case-cohort study sample (iPSYCH2012), hereafter referred to as iPSYCH sample 19 .

Details on the iPSYCH sample have been described previously 19 . In brief, the iPSYCH sample is nested within the entire Danish population of singleton births born to known mothers between 1981 and 2005 (study base: 1 472 762 individuals), who were alive and resided in Denmark on their first birthday 19 . The iPSYCH sample contains five cohorts of a combined total of 57 377 individuals with at least one diagnosis of one of five selected mental disorders, further referred to as case cohorts, i. e. affective/mood disorder(depression), attention-deficit/hyperactivity disorder (ADHD), autism, bipolar affective disorder (BD) and schizophrenia (SZ), and a representative, randomly selected cohort of the general population of 30 000 individuals corresponding to 2.04% of the study base. The members of the population-based cohort are representative of the entire Danish population born between 1981 and 2006, and are at risk of developing the disorder of interest during follow-up.

### Data sources

The iPSYCH sample is linked via the anonymized personal identification number, since birth or immigration, to drug prescription data and clinical and socio-demographic information from several Danish national registers, including *i* ) the Danish Civil Registration System (CPR) including information since 1968 on e. g. birth registration, vital status and citizenship 20 ; *ii* ) the Danish National Prescription Registry including information on all prescription drugs dispensed at pharmacies since 1994, including e. g. the anatomical therapeutic chemical (ATC) classification code, date and quantity of dispensed drugs 21 ; *iii* ) the Danish National Patient Registry including inpatient care information in Denmark since 1977 and outpatient care information since 1995 22 ; *iv* ) the Danish Psychiatric Central Research Register including e. g. diagnosis at and dates of admission and discharge of patients treated at psychiatric departments in Denmark since 1969 23 ; and *v* ) the Danish Register of Causes of Death including cause-specific mortality statistics, with computerized individual records since 1970 24 .

### Study population and study period

**Fig. 1:**
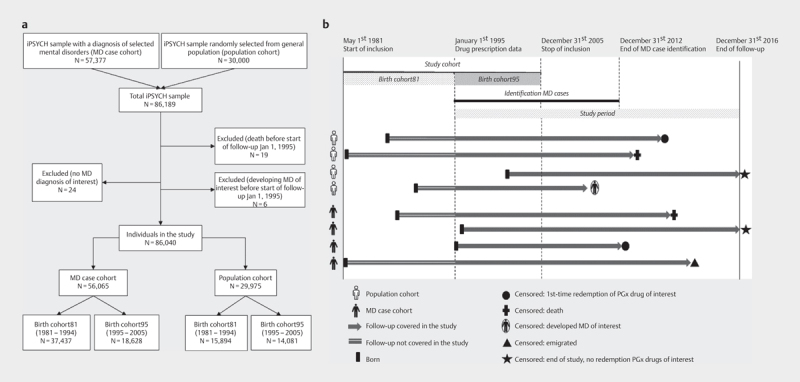
An overview of study sample selection, cohorts and follow-up . Panel **a** shows the selection of the iPSYCH sample in the study and panel **b** shows the overview of the study cohorts, study period and follow-up. The timeline in panel B shows the study cohort (1981-2005), comprising birth cohort81 (1981–1994) and birth cohort95 (1995–2005). The study period was from 1995 to 2016. The cohort was divided into two birth cohorts because prescription information was only available since 1995 and we did not have complete prescription information for individuals born between 1981 and 1994. The Danish Psychiatric Central Research Register contains registrations of contact moments until December 31 ^st^ , 2012, which is the latest date of defining individuals as MD cases for the case cohorts.

An overview of the study design and the included individuals with mental disorders and the population sample is shown in Fig. 1 , **panel a** . The study period was from January 1 ^st^ , 1995 until December 31 ^st^ , 2016. As prescription drug information from birth was not available for individuals born between 1981 and 1994, and to assess life-time exposure to prescription drugs, we created two birth cohorts including birth cohort81 born in 1981–1994 and birth cohort95 born in 1995–2005 (with life-time prescription drug exposure). The follow-up time of the individuals started in 1995 (or at birth after 1995), and ended at the date of emigration, death, or December 31 ^st^ , 2016, whatever came first ( Fig. 1 , **panel b** ). Individuals of the population cohort developing mental disorders during follow-up were censored from the population sample at the date of diagnosis. If these individuals had received a diagnosis of one of the included mental disorders before December 31, 2012, they were, by study design, included in the case cohorts. These individuals contributed observation time and drug use in both cohorts prior to their diagnoses but accounted only for a small proportion of the population cohort due to low incidence rates 37 . Thus, in this study, the population-based cohort represents the part of the Danish population without the selected psychiatric disorders diagnosed at psychiatric hospitals.

### Pharmacogenetic drugs of interest

The international Clinical Pharmacogenetics Implementation Consortium (CPIC) and the Dutch Pharmacogenetics Working Group (DPWG) execute literature reviews on PGx and provide peer-reviewed, evidence-based, updatable and detailed PGx guidelines 2 3 4 . National Danish PGx guidelines do not exist. CPIC and DPWG recommendations partially overlap with labelling recommendations of drugs, such as pimozide and warfarin regarding PGx testing, but PGx testing, in general, is not routinely/widely integrated in Danish clinical practice. We combined information from both CPIC and DPWG PGx guidelines and identified 69 drugs for which actionable PGx recommendations were provided until February 2020 (i. e. actionable PGx drugs, **Supplement Table 1** ) 25 26 . The list includes drugs from the following drug classes: anaesthetics, antibiotics, analgesics, anti-cancer drugs, anticoagulants, cardiovascular drugs, proton-pump inhibitors (PPIs) and psychotropic drugs. Of the 69 PGx drugs, 20 drugs (29%) have more than one actionable DGI, and thus have more than one actionable PGx guideline. Seven drugs have DGIs related to both *CYP2C19* and *CYP2D6* , three drugs to *TPMT* and *NUDT15* , seven drugs to *RYR1* and *CACNA1S* , one drug to *HLA-A* and *HLA-B* , one drug to *CYP2C9* and *HLA-B* and one drug to *VKORC1* , *CYP2C9* and *CYP4F2* ( **Supplement Table 1** ). We identified the use of actionable PGx using their ATC classification codes from the Danish National Prescription Registry ( **Supplement Table 1** ). The retrieved data thus describes drug prescriptions redeemed by the patients at community pharmacies, but the terms ‘drug use’ and ‘drug users’ are applied as well in this study.

### Data accessibility

The iPSYCH study was approved by the Danish Scientific Ethics Committee (EC: 1-10-72-287-12), the Danish National Board of Health (Sundhedsdatastyrelsen, SDS, FSEID 1999) and the Danish Data Protection Agency (Journal number 2015-57-0002, 62908, umbrella permission Aarhus University). All data is stored at Statistics Denmark and was available in an anonymous form, by remote online access, with special permission in compliance with the Danish Data Privacy Act.

### Statistical analyses

We have presented measures of drug use e. g. incidence rates and prevalence, means, standard deviations, separately for two birth cohorts, males and females, and mental disorders case cohorts and the population cohort. We divided the number of users with at least one prescription of a respective PGx drug by the number of total underlying person-years (PY) during follow-up (incidence rates) and by the number of total underlying individuals at the beginning of follow-up (prevalence). The mean age of first-time PGx drug use and the mean number of prescribed different PGx drugs were tested with a t-test to examine whether the mean differences between males and females were statistically significant. A p-value <0.05 was considered statistically significant. We used SAS %Lexis macro to calculate incidence rates 27 . Individuals with mental disorders might have received a first actionable psychotropic PGx drug prescription prior to their diagnosis of mental disorders, hence, we have reported descriptive statistics for those individuals who used PGx drugs prior to their first psychiatric hospital diagnosis within each cohort. The number of different DGIs per individual was calculated as the sum of all unique DGIs during follow-up. Concomitant drug use was considered if two or more different drugs had at least one day of overlap of their drug prescriptions. To assess if panel-based testing is more favourable than single-gene testing, we identified the number of prescribed PGx drugs which have more than one actionable DGI, and the total number of users for those drugs.

Due to a restriction from Statistics Denmark and the General Data Protection Regulations, data can only be reported if the number of individuals contributing to aggregated measures exceeds four, which can result in the grouping of data. For sub-analyses to avoid too few counts in individual categories, we grouped mental disorders into mental disorders (A) including predominantly childhood onset disorders and (B) including predominantly adult-onset disorders. All data were processed and analysed using SAS statistical software version 9.4 (SAS Institute Inc, Cary, NC USA) and proportions were compared using MedCalc for Windows, version 19.4 (MedCalc Software, Ostend, Belgium).

## Results

Table 1 shows the characteristics for both the randomly selected population cohort (N=29 975) and the combined mental disorders case cohorts (N=56 065) born between 1981–2005 in Denmark, further divided into the two birth cohorts. Males and females were equally distributed in all cohorts, except in the mental disorders case birth cohort95, which included more males due to a higher prevalence of the young-onset of ADHD and autism in males. Individuals can have a diagnosis of more than one mental disorder and can therefore contribute to several mental disorder case cohorts. During the total follow up of 1 664 266 PY (average of 19.3 years), 115 individuals (0.13%) died and 3260 individuals (3.8%) emigrated (0.19 and 6.1% in the population cohort, and 0.10 and 2.6% in the mental disorders cohorts, respectively).

**Table:** **Table 1** Characteristics of study sample.

	Birth cohort81 (1981–1994) ^**†**^ Total=53,331				Birth cohort95 (1995–2005) ^**†**^ Total=32 709			
	Population (N=15 894)		MD case cohorts (N=37 437)		Population (N=14 081)		MD case cohorts (N=18 628)	
	N	(%)	N	(%)	N	(%)	N	(%)
**Age in groups (y)**								
<18	-	-	-	-	8876	(63.0)	9705	(52.1)
18-23	2636	(16.6)	5605	(15.0)	5205	(37.0)	8923	(47.9)
24-29	7349	(46.2)	17 494	(46.7)	-	-	-	-
30-35	5909	(37.2)	14 338	(38.3)	-	-	-	-
**Sex**								
Female	7817	(49.2)	19 619	(52.4)	6866	(48.8)	4971	(26.7)
Male	8077	(50.8)	17 818	(47.6)	7215	(51.2)	13 657	(73.3)
**Ethnicity** ^**‡**^								
Africa	65	(0.4)*	73	(0.2)	218	(1.5)*	192	(1.0)
Asia	176	(1.1)*	146	(0.4)	206	(1.5)*	143	(0.8)
Australia/Greenland/N.&S.America/Unknown	16	(0.1)*	16	(0.0)	14	(0.1)	14	(0.1)
Denmark	13 861	(87.2)*	33 001	(88.2)	11 294	(80.2)*	15 638	(83.9)
Europe	308	(1.9)*	403	(1.1)	360	(2.6)*	271	(1.5)
Middle East	145	(0.9)*	161	(0.4)	339	(2.4)*	256	(1.4)
Mixed	1298	(8.2)*	3601	(9.6)	1609	(11.4)	2088	(11.2)
Scandinavia	25	(0.2)*	36	(0.1)	41	(0.3)*	26	(0.1)
**Region in Denmark**								
Capital Region	4629	(29.1)	10 925	(29.2)	4609	(32.7)	7011	(37.6)
Middle Jutland	3580	(22.5)	8764	(23.4)	2991	(21.2)	3407	(18.3)
North Jutland	2178	(13.7)	4626	(12.4)	1863	(13.2)	2647	(14.2)
Southern Denmark	1804	(11.4)	3180	(8.5)	1453	(10.3)	1257	(6.7)
Zealand	3703	(23.3)	9942	(26.6)	3165	(22.5)	4306	(23.1)
**Diagnosis with MD** ^**§**^								
ADHD	271	(1.7)	9402	(25.1)	292	(2.1)	10 303	(55.3)
Affective disorders	558	(3.5)	23 840	(63.7)	72	(0.5)	2228	(12.0)
Bipolar disorder	50	(0.3)	2014	(5.4)	<5	(0.0)	87	(0.5)
Depression	516	(3.2)	22 025	(58.8)	67	(0.5)	2085	(11.2)
Autism	134	(0.8)	6548	(17.5)	223	(1.6)	9564	(51.3)
Schizophrenia	140	(0.9)	4271	(11.4)	8	(0.1)	214	(1.1)

† Birth cohort81 includes individuals born in 1981-1994 and birth cohort95 includes individuals born in 1995-2005; ^‡^ Ethnicity defined based on parental place of birth as described by Pedersen et al. 18 Europe means countries other than Denmark/Scandinavia and Scandinavia means only Norway, Sweden, Finland, and Iceland. If one parent was born outside of Denmark, that region was used. If both parents were born outside of Denmark, but in different regions, mixed was used. ^§^ Summing the percentages of individual disorders might add up to more than 100%, as individuals can carry multiple diagnoses; *Significant difference at a p-value= <0.05. Presented are the characteristics of individuals in the population cohort and combined MD case cohort assessed in 2016. The data is presented in two separate birth cohorts. Data with a number below 5 is presented as ‘<5’ for privacy safety reasons. Abbreviations: ADHD: attention-deficit/hyperactivity disorder, MD: mental disorders.

### Incidence rates of pharmacogenetic drug use

Of the 69 drugs with actionable PGx recommendations, we identified 45 PGx drugs for which prescriptions were redeemed at community pharmacies, 10 of which were used by less than five users each, but were included in further analyses of cumulative or combined use ( Table 2 ). In the population cohort, the highest incidence rates of PGx drug use per 10 000 PY were recorded for oestrogens (429 in females), the analgesics codeine (27 in males and 55 in females) and tramadol (34 in males and 45 in females), followed by PPIs (lansoprazole, omeprazole and pantoprazole) and the antidepressant citalopram ( Table 2 ). In the mental disorders case cohorts, the incidence rates of any of the PGx drugs use per 10 000 PY were higher compared with the population cohort, in particular, psychotropic drugs matching their main indications, e. g. atomoxetine (160 in males and 188 in females) in ADHD, citalopram (248 in males and 306 in females) in depression, aripiprazole (229 in males and 316 in females) in SZ, and lamotrigine (211 in males and 330 in females) in BD. Individuals in the mental disorders case cohorts were prescribed at least one psychotropic PGx drug before their first mental disorders diagnosis with proportions of 50.4% (ADHD), 38.2% (autism), 86.9% (SZ), 86.1% (BD), and 83.3% (depression).

**Table:** **Table 2** Incidence rates of PGx prescription drug use per 10 000 PY of the iPSYCH sample, by population cohort and individual MD case cohorts and sex.

Drug name		Population (N=29 975) N/10 000 PY		ADHD (N=19 705) N/10 000 PY		Autism (N=16 112) N/10 000 PY		Bipolar disorder (N=2101) N/10 000 PY		Depression (N=24 110) N/10 000 PY		Schizophrenia (N=4485) N/10 000 PY		PGx DGI
		Females	Males	Females	Males	Females	Males	Females	Males	Females	Males	Females	Males	
Allopurinol		-	-	-	-	-	-	1	1	1	1	2	1	HLA-B ^a,b^
Amitriptyline	P	6	2	11	5	6	3	21	10	24	16	23	9	CYP2D6 ^a,b^ , CYP2C19 ^a^
Aripiprazole	P	1	1	47	31	56	38	143	116	51	54	316	229	CYP2D6 ^b^
Atomoxetine	P	2	4	188	160	49	51	32	57	23	40	27	38	CYP2D6 ^a,b^
Atorvastatin		1	-	1	1	1	1	2	3	2	3	5	7	SLCO1B1 ^b^
Azathioprine		2	2	3	2	2	2	4	3	4	4	4	3	TPMT ^a,b^ , NUDT15 ^a,b^
Carbamazepine	P	1	1	5	7	10	6	9	18	3	6	8	10	HLA-A ^a,b^ , HLA-B ^a,b^
Citalopram	P	31	14	135	51	84	42	314	214	306	248	274	185	CYP2C19 ^a,b^
Clomipramine	P	-	-	2	1	3	1	12	4	7	6	9	5	CYP2D6 ^a,b^ , CYP2C19 ^a,b^
Clopidogrel		-	-	-	-	-	-	1	2	1	-	2	-	CYP2C19 ^a,b^
Codeine		55	27	89	38	48	24	125	76	128	67	128	62	CYP2D6 ^a,b^
Doxepin	P	-	-	-	-	-	-	-	1	-	1	-	-	CYP2D6 ^a,b^ , CYP2C19 ^a^
Escitalopram	P	9	4	46	18	33	15	145	105	113	101	124	87	CYP2C19 ^a,b^
Oestrogens		429	-	484	-	292	-	735	-	716	-	600	-	F5/FvL ^b^
Flucloxacillin		10	10	22	16	17	14	14	20	20	15	28	17	HLA-B ^b^ HLA-B ^b^
Fluvoxamine	P	-	-	6	-	-	-	1	1	1	-	1	-	CYP2D6 ^a^
Haloperidol	P	-	-	2	2	4	1	7	12	2	2	20	12	CYP2D6 ^b^
Imipramine	P	1	1	2	1	2	1	3	4	3	4	8	6	CYP2C19 ^a,b^ , CYP2D6 ^a,b^
Lamotrigine	P	6	4	65	25	51	23	330	211	81	49	116	41	HLA-B ^b^
Lansoprazole		35	20	84	36	39	21	107	60	99	64	129	74	CYP2C19 ^b^
Metoprolol		7	2	9	5	9	3	15	10	17	9	22	11	CYP2D6 ^b^
Nortriptyline	P	2	1	8	3	5	2	33	23	25	22	20	13	CYP2D6 ^a,b^ , CYP2C19 ^a^
Omeprazole		38	20	70	31	50	23	86	41	86	53	107	55	CYP2C19 ^b^
Ondansetron		4	1	7	1	3	2	13	2	10	2	10	2	CYP2D6 ^a^
Oxcarbazepine	P	2	2	5	6	11	8	6	6	3	4	8	5	HLA-B ^a,b^
Pantoprazole		35	21	82	39	45	24	108	69	98	68	137	85	CYP2C19 ^b^
Paroxetine	P	3	2	16	6	11	6	26	19	27	23	32	24	CYP2D6 ^a,b^
Pimozide	P	-	1	3	6	2	4	1	4	2	4	6	8	CYP2D6 ^b^
Risperidone	P	2	3	60	63	73	69	111	110	53	70	237	226	CYP2D6 ^b^
Sertraline	P	22	12	132	56	133	64	217	148	226	179	235	142	CYP2C19 ^a,b^
Simvastatin		1	1	4	3	4	2	12	10	7	9	22	23	SLCO1B1 ^a,b^
Tramadol		45	34	106	57	41	22	148	107	147	104	163	86	CYP2D6 ^b^
Venlafaxine	P	10	4	66	24	36	15	158	104	152	128	146	88	CYP2D6 ^b^
Warfarin		2	1	2	1	1	1	4	3	3	2	8	2	VKORC1 ^a,b^ , CYP2C9 ^a,b^ , CYP4F2
Zuclopenthixol	P	-	-	6	5	7	3	21	19	8	8	59	46	CYP2D6 ^b^

Incidence rates of PGx drug prescriptions in the population cohort and for the five individual MD case cohorts are shown as number of users per 10,000 PY of the total observed follow-up time. Ten drugs though identified in the study population, but not included in this table are flecainide, fluorouracil, mercaptopurine, phenprocoumon, phenytoin, propafenone, tacrolimus, tamoxifen, trimipramine and voriconazole because these drugs were used by less than 10 individuals in the total population or MD case cohorts. ^a^ Actionable PGx guideline from CPIC. ^b^ Actionable PGx guideline from DPWG. Abbreviations: ADHD: attention-deficit/hyperactivity disorder, ATC: Anatomical Therapeutic Chemical, CPIC: Clinical Pharmacogenetics Implementation Consortium, DPWG: Dutch Pharmacogenetics Working Group, DGI: drug-gene interaction, MD: mental disorders, PY: person-years, P: psychotropic drug, PGx: pharmacogenetics.

### Prevalence of pharmacogenetic drug use

In the population birth cohort81, besides oestrogens used by 80.7% of females, tramadol (11.7%), codeine (11.3%) and lansoprazole (7.7%) were the most frequently prescribed PGx drugs. In the younger population birth cohort95, the most used PGx drugs besides oestrogens were omeprazole (3.1%), codeine (2.7%) and pantoprazole (2.4%) ( **Supplement Table 2** ). In general, the prevalence of drug users was higher in both the mental disorders case birth cohorts compared with the population cohorts. In the mental disorders case birth cohort81, the most prevalent prescribed PGx drugs besides oestrogens were citalopram (39.3%), sertraline (30.7%) and tramadol (23.5%). In the mental disorders case birth cohort95, the most prevalent prescribed PGx drugs were atomoxetine (17%), oestrogens (12.8%) and sertraline (10%) ( **Supplement Table 3** ).

### Age of first-time pharmacogenetic drug use

Age of first-time PGx drug use (life-time use) differed between sex in all cohorts born since 1995 ( Table 3 , **upper part** ). The mean age of starting a PGx drug for the first-time ranged from 11.6–15.0 years for males and 13.1–15.2 years for females. The youngest individuals starting a first-time PGx drug on average were 11.6 years old males with ADHD or autism, compared with nearly 13 years of age in females with ADHD or autism.

**Table:** **Table 3** Age at first-time PGx drug use and mean number of different PGx drugs of the iPSYCH sample, by birth and population and MD cohorts and sex.

Age at first-time PGx drug use								
Cohort	Birth cohort81 (1981–1994) ^**†**^				Birth cohort95 (1995–2005) ^**†**^			
	Females (N=25 726)		Males (N=16 005)		Females (N=6299)		Males (N=5949)	
	Mean	±SD	Mean	±SD	Mean	±SD	Mean	±SD
Population	17.8	3.5	21.6	5.7	15.2	3.3	12.9	5.1
ADHD	16.1	3.0	19.0	4.9	13.2	3.6	11.6	4.1
Autism	16.2	4.0	17.4	5.4	13.1	4.3	11.6	4.4
Bipolar disorder ^¥^	17.0	2.8	20.0	4.2	15.0	1.8	14.1	3.9
Depression	16.8	2.8	19.9	4.2	14.7	2.6	13.8	3.5
Schizophrenia ^¥^	16.9	2.9	19.8	4.1	14.3	2.9	15.0	2.8
Mean number of different PGx drugs prescribed								
Cohort	Birth cohort81 (1981–1994) ^**†**^				Birth cohort95 (1995–2005) ^**†**^			
	Females (N=25 726)		Males (N=16 005)		Females (N=6299)		Males (N=5949)	
	Mean	±SD	Mean	±SD	Mean	±SD	Mean	±SD
Population	2.0	1.4	1.6	1.1	1.4	0.8	1.2	0.6
ADHD	4.4	2.5	2.8	2.0	2.1	1.3	1.6	1.0
Autism	3.5	2.3	2.4	1.6	2.0	1.2	1.7	1.0
Bipolar disorder	5.3	2.6	3.6	2.2	3.4	1.8	2.3	1.3
Depression	4.4	2.4	3.1	2.0	2.6	1.5	2.0	1.2
Schizophrenia	5.6	2.9	3.6	2.2	3.5	1.6	2.2	1.1

† Birth cohort81 includes individuals born between 1981 and 1994, birth cohort95 includes individuals born between 1995 and 2005. ¥ The differences between males and females in all cohorts are statistically significant, except for the birth cohort95 with bipolar disorder or schizophrenia. Mean age of drug users at their first-time PGx drug prescription and the mean of the number of PGx drugs prescribed in the population and for five individual MD diagnoses are presented. The table is split based on birth cohorts, as we do not have life-time drug use available for individuals in birth cohort81. Individuals who were not prescribed any PGx drug prior to death or data capping are not included in this table. Abbreviations: MD: mental disorders; ADHD: attention-deficit/hyperactivity disorder; iPSYCH: Integrative Psychiatric Research (iPSYCH) consortium, SD: standard deviation.

### Multiple (different) pharmacogenetic drugs per user

Individuals used on average more than one PGx drug over a maximum follow-up time of 22 years ( Table 3 , **lower part** ) with sex and birth cohort differences in both the population and mental disorders case cohorts. Overall, higher means of different PGx drugs were seen in the mental disorders case cohorts compared with the population cohorts, with maximum means of more than four different PGx drugs in females of the mental disorders birth cohort81.

The prevalence of individuals using different PGx drugs (0 – > 9) is mentioned in Table 4 . Within the population cohort, 23.1% of males and 65.4% of females used one or more PGx drugs during the follow-up time. Among individuals of the mental disorders case birth cohorts combined, 56.2% of males and 84.9% of females with ADHD, 43.8% and 72.3% with autism, 87% and 96% with BD, 86.5% and 96.9% with depression, and 87.3% and 97.2% with SZ, respectively, used one or more PGx drugs during the follow-up time. The highest prevalence of individuals using more than three different PGx drugs exceeded 80% in females with BD or SZ. The highest prevalence of individuals using six or more PGx drugs was approximately 40% in females with BD or SZ.

**Table:** **Table 4** Prevalence of individuals of the iPSYCH sample with increasing number of different PGx drugs, by population and MD cohorts and sex.

No. of different PGx drugs	Population				ADHD				Autism				Bipolar disorder				Depression				Schizophrenia			
	Female		Male		Female		Male		Female		Male		Female		Male		Female		Male		Female		Male	
	(N=14 683)		(N=15 292)		(N=5289)		(N=14 416)		(N=3525)		(N=12 587)		(N=1292)		(N=809)		(N=16 476)		(N=7634)		(N=2083)		(N=2402)	
	N	(%)	N	(%)	N	(%)	N	(%)	N	(%)	N	(%)	N	(%)	N	(%)	N	(%)	N	(%)	N	(%)	N	(%)
0	5081	(34.6)	11 766	(76.9)	799	(15.1)	6317	(43.8)	976	(27.7)	7079	(56.2)	52	(4.0)	105	(13.0)	518	(3.1)	1028	(13.5)	58	(2.8)	305	(12.7)
1	5409	(36.8)	2374	(15.5)	1029	(19.5)	3662	(25.4)	854	(24.2)	2641	(21.0)	76	(5.9)	125	(15.5)	1430	(8.7)	1600	(21.0)	87	(4.2)	377	(15.7)
2	2263	(15.4)	732	(4.8)	822	(15.5)	1878	(13.0)	588	(16.7)	1383	(11.0)	125	(9.7)	154	(19.0)	2534	(15.4)	1565	(20.5)	201	(9.6)	398	(16.6)
3	1002	(6.8)	239	(1.6)	712	(13.5)	1097	(7.6)	397	(11.3)	732	(5.8)	144	(11.1)	122	(15.1)	2949	(17.9)	1222	(16.0)	262	(12.6)	426	(17.7)
4	489	(3.3)	114	(0.7)	590	(11.2)	593	(4.1)	289	(8.2)	366	(2.9)	210	(16.3)	99	(12.2)	2684	(16.3)	887	(11.6)	289	(13.9)	320	(13.3)
5	228	(1.6)	37	(0.2)	448	(8.5)	358	(2.5)	182	(5.2)	192	(1.5)	189	(14.6)	85	(10.5)	2116	(12.8)	558	(7.3)	293	(14.1)	204	(8.5)
6	125	(0.9)	19	(0.1)	355	(6.7)	241	(1.7)	102	(2.9)	107	(0.9)	141	(10.9)	49	(6.1)	1600	(9.7)	341	(4.5)	247	(11.9)	152	(6.3)
7	45	(0.3)	11 ^**†**^	(0.0)	177	(3.3)	118	(0.8)	54	(1.5)	43	(0.3)	116	(9.0)	35	(4.3)	1028	(6.2)	203	(2.7)	186	(8.9)	92	(3.8)
8	17	(0.1)			158	(3.0)	76	(0.5)	34	(1.0)	26	(0.2)	93	(7.2)	13	(1.6)	722	(4.4)	123	(1.6)	150	(7.2)	62	(2.6)
9	16	(0.1)			77	(1.5)	42	(0.3)	20	(0.6)	12	(0.1)	65	(5.0)	9	(1.1)	387	(2.3)	65	(0.9)	110	(5.3)	37	(1.5)
>9	8	(0.1)			122	(2.3)	34	(0.2)	29	(0.8)	6	(0.0)	81	(6.3)	13	(1.6)	508	(3.1)	42	(0.6)	200	(9.6)	29	(1.2)

^**†**^ This number is grouped with data from >7 different PGx drugs, due to data otherwise being <5. The number of individuals who were prescribed different numbers of PGx drugs in the population cohort and for five individual MD case cohorts over a mean follow-up time of 19-21 years is shown. Mean (±SD) follow-up times were 19 years in the male population (±4.1), male patients with ADHD (±3.7) and patients with autism (±3.8), 20 years in the female population (±4) and female patients with ADHD (±3.5) and 21 years in patients with BD (F ±3.6, M ±3.4), depression (F ±3.1, M ±3) and SZ (F ±3, M ±3.1). Abbreviations: MD: mental disorders; ADHD: attention-deficit/hyperactivity disorder; iPSYCH: Integrative Psychiatric Research, PGx: pharmacogenetics, SD: standard deviation, F: females, M: males.

### Pharmacogenetic drug use relevant to panel-based testing and combinatorial PGx

Of the 39 PGx drugs, nine drugs (23%) with more than one actionable DGI, including genes coding for *CYP2D6* , CYP2C19, HLA-A, HLA-B, CYP2C9, CYP4F2 or VKORC1 were used by 3.9% of the PGx drug users of the population and 9.7% of the PGx drug users of the combined mental disorders case cohorts.

The prevalence of individuals using different PGx drugs of different DGIs at any time during the follow-up was 43% in females and 25.3% in males in the population cohort ( Table 5 ). In individuals with mental disorders, these numbers ranged between 39.6% in males with autism and 94.1% in females with SZ. The involved DGIs are listed in the legend of Table 5 .

**Table:** **Table 5** Number of individuals using PGx drugs and the relation with different genes of the iPSYCH sample, by populations and MD cohorts and sex.

No. of different types of genes	Population				ADHD				Autism				Bipolar disorder				Depression				Schizophrenia			
	Female		Male		Female		Male		Female		Male		Female		Male		Female		Male		Female		Male	
	(N=9602)		(N=3526)		(N=4490)		(N=8099)		(N=2549)		(N=5508)		(N=1240)		(N=704)		(N=15 958)		(N=6606)		(N=2025)		(N=2097)	
	N	(%)	N	(%)	N	(%)	N	(%)	N	(%)	N	(%)	N	(%)	N	(%)	N	(%)	N	(%)	N	(%)	N	(%)
1	5473	(57.0)	2635	(74.7)	1113	(24.8)	4720	(58.3)	960	(37.7)	3328	(60.4)	83	(6.7)	182	(25.9)	1695	(10.6)	2382	(36.1)	120	(5.9)	600	(28.6)
2	2703	(28.2)	765	(21.7)	1237	(27.6)	2682	(33.1)	821	(32.2)	1768	(32.1)	207	(16.7)	269	(38. 2)	4575	(28.7)	3258	(49.3)	458	(22.6)	1127	(53.7)
3	1237	(12.9)	98	(2.8)	1538	(34.3)	536	(6.6)	544	(21.3)	334	(6.1)	404	(32.6)	217	(30.8)	6991	(43.8)	793	(12.0)	942	(46.5)	291	(13.9)
4	142	(1.5)	21	(0.6)	532	(12.1)	145	(1.8)	185	(7.3)	78 ^**†**^	(1.4)	493	(39.8)	28	(4.0)	2345	(14.7)	144	(2.2)	399	(19.7)	68	(3.2)
5	47 ^**†**^	(0.5)	7	(0.2)	58	(1.3)	16 ^**†**^	(0.2)	39 ^**†**^	(1.5)	-	-	39	(3.2)	8 ^**†**^	(1.1)	291	(1.8)	29 ^**†**^	(0.4)	84	(4.2)	11	(0.5)
≥6	-	-	-	-	12 ^**†**^	(0.3)	-	-	-	-	-	-	14 ^**†**^	(1.1)	-	-	61 ^**†**^	(0.4)	-	-	22 ^**†**^	(1.1)	-	-

^**†**^ This number is grouped with data from the immediate cell below, due to data otherwise being <5. The numbers of individuals are shown for different numbers of genes involved in the prescribed PGx drugs in the population and for five individual MD diagnoses over a mean follow-up time of 20-22 years. Mean (±SD) follow-up times were 20 years in the population (F ±3, M ±3.1), patients with ADHD (F ±2.7, M ±3) and autism (F ±2.7, M ±3), 21 years in patients with BD (F ±2.5, M ±2) and female patients with depression (±2.2), and 22 years in male patients with depression (±1.7) and patients with SZ (F ±2, M ±1.7). Percentages are calculated only among PGx drugs users. Genes linked to drug use per cohort: Population (CYP2C19, CYP2D6, CYP4F2, F5/FvL, HLA-A, HLA-B, NUDT15, SLCO1B1, TPMT, VKORC1, CYP3A5); ADHD (CYP2C19, CYP2D6, CYP4F2, F5/FvL, HLA-A, HLA-B, NUDT15, SLCO1B1, TPMT, VKORC1); Autism (CYP2C19, CYP2D6, CYP4F2, F5/FvL, HLA-A, HLA-B, NUDT15, SLCO1B1, TPMT, VKORC1, CYP3A5*, DPYD* (* only in males); BD (CYP2C19, CYP2D6, CYP4F2, F5/FvL*, HLA-A, HLA-B, NUDT15, SLCO1B1, TPMT, VKORC1 (* only in females); MDD (CYP2C19, CYP2D6, CYP4F2, F5/FvL, HLA-A, HLA-B, NUDT15, SLCO1B1, TPMT, VKORC1); SZ (CYP2C19, CYP2D6, CYP4F2, F5/FvL, HLA-A, HLA-B, NUDT15, SLCO1B1, TPMT, VKORC1). Abbreviations: MD: mental disorders; ADHD: attention-deficit/hyperactivity disorder; iPSYCH: Integrative Psychiatric Research, PGx: pharmacogenetics, SD: standard deviation; F: females; M: males.

Concerning combinatorial PGx, the prevalence of individuals who used concomitantly different PGx drugs affected by different DGIs ranged between 24.4% of the individuals without mental disorders, 41.3% of individuals with autism or ADHD to 69.2% of individuals with BD, SZ or depression ( Table 6 ). In over 80% of these users, the PGx drugs were linked to two different DGIs, in 8.1% to three, and in 1.1% of these users, to four DGIs.

**Table:** **Table 6** Number of individuals using concomitant PGx drugs and affected by different genes.

Cohorts ^†^	Number of individuals with concomitant drugs N (%) ^‡,§^			One gene N (%) ^¶^			Two genes N (%) ^¶^			Three genes N (%) ^¶^			Four genes N (%) ^¶,ͳ^		
	Total	Female	Male	Total	Female	Male	Total	Female	Male	Total	Female	Male	Total	Female	Male
**Population** (N=13 128) (F=9602/M=3526)	3199 (24.4)	2727 (28.4)	472 (13.4)	728 (22.8)	501 (18.4)	227 (48.1)	2887 (90.2)	2589 (95.0)	298 (63.1)	198 (6.2)	162 (5.9)	36 (7.6)	36 (1.1)	29 (1.1)	7 (1.5)
**MD-A**^ψ^ (N=18 531) (F=6570/M=11 961)	7653 (41.3)	3818 (58.1)	3835 (32.1)	3800 (49.7)	1588 (41.6)	2212 (57.7)	6419 (83.9)	3587 (93.9)	2832 (73.8)	441 (5.8)	250 (6.5)	191 (5.0)	48 (0.6)	22 (0.6)	26 (0.7)
**MD-B**^χ^ (N=25 355) (F=17 255/M=8100)	17 546 (69.2)	13 349 (77.4)	4197 (51.8)	8499 (48.4)	6083 (45.6)	2416 (57.6)	16 137 (92.0)	12 769 (95.7)	3368 (80.2)	1418 (8.1)	1205 (9.0)	213 (5.1)	148 (0.8)	116 (0.9)	32 (0.8)

^†^ Only those individuals who had a history of using PGx drugs were included; ^‡^ The percentages are estimated out of the total number of individuals in each cohort; ^§^ Concomitant drug use implies at least two different drugs overlapping for at least one day; ^¶^ The percentages are estimated out of the number of individuals using concomitant PGx drugs; ^ͳ^ Only a few individuals used concomitant drugs metabolized by five genes (all less than five observations); ^ψ^ MD-A includes ADHD and autism; ^χ^ MD-B includes schizophrenia, bipolar disorder and depression. The number of individuals using PGx drugs concomitantly is shown, followed by the number of involved genes related to the PGx drugs. The mean (±SD) days of overlap were 676 (±2,300); 2,584 (7,510) and 3,045 (7,732) for the population cohort, MD-A MD-B case cohorts, respectively. Abbreviations: MD: mental disorders; ADHD: attention-deficit/hyperactivity disorder; SD: standard deviation; F: female; M: male.

## Discussion

This is the first population-based PGx drug utilization study in 86 040 young people with and without mental disorders in Denmark describing (life-time) incident use of 39 of the 69 actionable PGx drugs according to international guidelines. We found that, by the age of 35 years, at least one actionable PGx had been used by up to 97% of individuals with mental disorders, i. e. with SZ, and by 65% of females and 23% of males of the population cohort (without mental disorders). In individuals with mental disorders, the most frequent actionable PGx drugs corresponded to their psychiatric indications, i. e. atomoxetine in ADHD, citalopram in depression, aripiprazole in SZ, and lamotrigine in BD, related to DGIs involving *CYP2D6* , *CYP2C19* , and *HLA-B* . Moreover, the high use of oestrogens in oral contraceptives related to Factor V Leiden (FvL), the weak opioid analgesics codeine and tramadol, the PPIs lansoprazole, omeprazole, and pantoprazole, and citalopram in both the mental disorders and population cohorts also related to the CYP2D6, CYP2C19, and HLA-B indicate the broad applicability of PGx testing in the general population. The first-time users of PGx drugs were as young as (mean age of) 11 years in males with ADHD. Panel-based testing including at least the most commonly identified DGIs could be applicable for 95% of females with SZ down to approximately 25% of males in the general population. Combinatorial PGx testing, considering several different drugs and different DGIs at the same time, could be relevant for up to 70% of individuals with mental disorders and 24% of the general population without mental disorders.

### Life-time incidence and prevalence of pharmacogenetic drug use

Recently several studies in different settings have investigated the incidence and prevalence of actionable PGx drug use, but they neither addressed life-time use or age at first PGx drug prescription nor were they conducted in unselected case cohorts with mental disorders or population-based, thus comparisons are hampered 7 11 12 13 14 15 28 29 30 . Still, the pattern of prevalence of PGx drug use in our study, e. g., the most frequent use of oestrogens, followed by codeine, tramadol, PPIs and citalopram, is similar to the patterns reported by a previous study in Denmark based on publically available prescription sales data of the general population by age 44 years in 2017 14 . In comparison with studies in other populations, our observations are in line with findings from the US, UK and the Netherlands applying similar actionable PGx drug criteria. In the US, Samwald et al. found in individuals below 40 years that weak opioids, PPIs, SSRIs, atomoxetine and selected antipsychotics were among the top 8 of incident PGx drugs 11 . In UK, Youssef et al. investigated sales data of overall prescriptions dispensed in 2018, where patients by age 39 years most commonly dispensed prescriptions for antidepressants, oral contraceptives, anti-infectives, and PPIs 10 . Of note, we found generally frequent use of oestrogens, i. e. oral contraceptives related to FvL, which is similar to frequencies of prescriptions reported from the UK and the Netherlands 10 13 . Although oestrogen-containing oral contraceptives are included in the uPGx panel of PGx drugs, which we applied in this study, it should be noted that oral contraceptives containing oestrogens are currently only considered actionable in females with a previous personal or family history of thrombosis or additional risk factors for thrombosis such as smoking, diabetes, and obesity according to DWPG 4 . This may lead to the impression that the number of women where PGx actions should be considered is inflated in our study, with 8647 female users of oestrogens in the population cohort, and 18908 female users with psychiatric disorders, and among those, an estimated 6.6% were heterozygous and 0.1% were homozygous carriers of FvL 31 32 . We did not assess additional risk factors, thus the actual number of females where PGx would be applicable is unknown. However, among females with psychiatric disorders considering oestrogen-containing oral contraceptives, PGx guidance could be considered applicable to a larger extent due to common (comorbid) conditions, including diabetes, obesity and smoking 33 34 35 . Moreover, it has been previously studied that establishing FvL testing in all women before initiating oral contraceptives is unfeasible due to costs and a low predictive value of FvL testing 32 . Now, considering the increasing utility of PGx in general and panel-based testing decreasing costs, the cost-benefit of FvL testing should be revisited for inclusion in core panels of actionable PGx tests and multifactorial treatment decisions.

While overall patterns of actionable PGx drugs were similar across the different cohorts, the prevalence of any of the investigated actionable PGx drugs was higher among people with mental disorders than in the population without these conditions. This is mainly due to the preponderance of psychotropic drugs among actionable PGx drugs matching the psychiatric disorders, but the more frequent use of PGx drugs such as analgesics and PPIs with indications for somatic conditions also indicates a higher burden of somatic disorders in younger people with mental disorders compared with their peers.

### Age of first pharmacogenetic drug use and timing of PGx testing

We found that the mean age of the earliest PGx drug use in the cohort born between 1995–2006 was 11 years in males and 13 years in females with ADHD or autism, and of 13 years in males and 15 years in females without mental disorders, suggesting earliest reactive prospective testing around these ages. We are not aware of any other study assessing life-time incident use of PGx drugs.

Considering pre-emptive PGx testing, which is unlike reactive prospective testing unrelated to a prescription of a PGx drug in the first place, our PGx drug prevalence findings indicate that pre-emptive testing could support pharmacological treatment decisions in 23% of males and 65% of females of the general population (without mental disorders) by the age of 35 years. In individuals with mental disorders, pre-emptive PGx could be applicable in, e. g. up to 87% of males and 97% of females. We further estimate that pre-emptive testing and test results could be applied for a mean of 3.1 PGx drugs in young individuals with mental disorders and a mean of 1.6 PGx drugs in young individuals without mental disorders by age of 35 years. Several other studies have investigated the potential of pre-emptive testing, but not based on life-time use, in different patient populations across disorders, age ranges, settings and study set-ups (e. g. follow-up times) leading to a wider range of 11.2% to 97% of individuals exposed to one or more PGx drugs over a 2–20 year follow-up period, thus are not directly comparable with our findings 11 28 36 37 38 .

### Panel-based and combinatorial pharmacogenetic testing

Our results indicate, as discussed earlier, the utility of panel-based testing due to the frequent use of several PGx drugs related to different DGIs. Panel-based testing in particular, of a core panel including variants of *CYP2D6* and *CYP2C19* has been endorsed by many PGx societies and implementation initiatives 14 15 30 39 . These efforts are now being further supported by a recent meta-analysis finding that plasma levels of various antidepressant and antipsychotic drugs are associated with CYP2D6/CYP2C19 genotype-predicted metabolizer status supporting genotype-based dosing recommendations and ultimately PGx testing in people with mental disorders initiating psychotropic treatment 40 .

An additional benefit of panel-based PGx testing is the opportunity to execute combinatorial PGx, which considers the effect of multiple variants in different genes for PGx-based dose adjustments. This is of importance when a single drug has multiple actionable DGIs or when multiple PGx drugs are used concomitantly. The substantial number (a third) of individuals affected by multiple DGIs and 24.4% (population) to 69.2% (BD, SZ, depression cohorts) using multiple PGx drugs concomitantly in our study indicates the potential benefit from combinatorial PGx. Multiple PGx drugs acting on the same enzyme affected by genetic variation leading to DDGIs and related PGx guided drug dosage recommendations are yet not provided in international public guidelines and rarely transparently in commercial combinatorial PGx tests but are under development 41 42 43 . In addition, the difference between the number of individuals using a drug and the number of individuals who require necessary action vary from<1% to 50% with divergent geno-phenotypes 14 , which is largely dependent on the combination of actionable PGx guidelines for geno- or phenotypes and frequencies of these geno- and phenotypes.

### Strengths and limitations

The strengths of the current study are that it presents data of a large population-based case-cohort of individuals with mental disorders and a population cohort representative for the entire Danish population of young age. The study has no bias in the selection of individuals, very little missing data and little loss to follow-up due to the registry-based study set-up using the Danish Civil Registration System. All these strengths allow a valid estimation of first-time as well as life-time PGx drug use in Denmark, and these estimates are likely to extrapolate to countries with similar drug utilization patterns and health care systems. This study has some limitations. First, a considerable number of individuals have no drug data registered from birth until 1995 solely affecting birth cohort81. By dividing the cohort into two birth cohorts, we were able to present PGx drug use from birth up to the end of the study for the remaining individuals born since 1995 (ages 11–21). This affects the interpretation of the age of first PGx drug use and the mean number of PGx drug use. We lack data of the youngest years of individuals in birth cohort81, with a maximum of the first 11 years of an individual born in 1984. This might result in a higher mean age for birth cohorts81 due to the left-truncation. We have data until a maximum age of 11 years for individuals born in 2005, thus individuals starting a first PGx drug at age 12 or older are not included in the calculations. This might result in a lower mean age for birth cohorts95 due to right-truncation. Yet, since we know the average age of onset of disease, we expect that the real answer, e. g., ADHD and autism lies close to the mean age identified in birth cohorts95, compared to the other disorders with a real age of first PGx drug use in between the results for birth cohorts 1 and 2 39 40 44 45 . Another limitation is that hospital-based data is missing from the drug registries, excluding some actionable PGx drugs such as anti-infectives and drugs used in anaesthesia. Moreover, we present a drug utilization study of current actionable PGx drugs. The results presented in this study might be affected in the future by changing trends in drug use or updated PGx guidelines, for example including new PGx drugs. Lastly, the iPSYCH population is rather homogeneous with 88% of Danish or European ancestry, 10% mixed ancestry, and only up to 2% of Non-European ancestry, partially due to the design of the study including individuals born in Denmark since 1981. Among the commonly used PGx drugs identified in the current study, this may affect estimates for the even greater utility of PGx testing of drugs affected by CYP2C19 variations or HLA-B variants, both of which are more frequent in individuals with Asian ancestry; or CYP2C9 variations in individuals with African ancestry and should be considered in PGx adjusted dosing recommendations of relevant drugs 46 47 .

In conclusion, PGx drugs are commonly used by young individuals, with more frequent PGx drug use among young individuals with mental disorders and females. PGx testing could be beneficial already at a very young age (adolescent). Panel-based PGx testing would be preferable over single-gene testing, based on the number of individuals using PGx drugs subsequently or concomitantly and the number of different drug-gene interactions involved.

## Data Availability Statement

The data that support the findings of this study are available from Statistics Denmark. Restrictions apply to the availability of these data, which were used under license for this study. Data are available in an anonymous form, by remote online access with the permission of Statistics Denmark, the National Centre for Register-based Research (NCRR) and the Centre for Integrated Register-Based Research at Aarhus University (CIRRAU).

FundingThe study was funded by unrestricted grants received by C. Gasse of the Alfred Benzon Foundation, Denmark, and NovoNordisk Foundation, Denmark (NNF17OC0029488) and by C. Lunenburg of the Lundbeck Foundation, Denmark (R322-2019-2404). The funders had no role in study design, data collection and analysis, decision to publish, or preparation of the manuscript.
