# Supplementary material for: Life-time Actionable Pharmacogenetic Drug Use: A Population-based Cohort Study in 86 040 Young People With and Without Mental Disorders in Denmark
Source: Pharmacopsychiatry. 2021 Nov 9;55(2):95–107. doi: 10.1055/a-1655-9500 (PMC8964272; doi:10.1055/a-1655-9500)
Supplement: Supplementary file 1 — Supplementary Material [file 10-1055-a-1655-9500-2021-03-1018.pdf]

Supplement Material

Life-time Data of Actionable Pharmacogenetic Drug Use: A Population-based Cohort Study in 86,040 Young People With and Without Mental Disorders in Denmark; Lunenburg *et al.*

Supplement Table 1. A list of drugs investigated in this study

Drug name	ATC code	PGx DGI
Abacavir	J05AF06	HLA-B [a,b]
Acenocoumarol	*B01AA07*	VKORC1 [b]
Allopurinol	M04AA01	HLA-B [a,b]
Amitriptyline	N06AA09, *N06CA01*	CYP2D6 [a,b], CYP2C19 [a]
Aripiprazole	N05AX12	CYP2D6 [b]
Atazanavir	J05AE08, J05AR15, *J05AR23*	UGT1A1 [a]
Atomoxetine	N06BA09	CYP2D6 [a,b]
Atorvastatin	C10AA05, *C10BX08, C10BX03*, C10BA05, *C10BX12, C10BX06, C10BX11, C10BX15*	SLCO1B1 [b]
Azathioprine	L04AX01	TPMT [a,b], NUDT15 [a,b]
Brexpiprazole	N05AX16	CYP2D6 [b]
Capecitabine	L01BC06	DPYD [a,b]
Carbamazepine	N03AF01	HLA-A [a,b], HLA-B [a,b]
Citalopram	N06AB04	CYP2C19 [a,b]
Clomipramine	N06AA04	CYP2D6 [a,b], CYP2C19 [a,b]
Clopidogrel	B01AC04	CYP2C19 [a,b]
Codeine	N02AJ07, *N02AJ08, N02AJ09,* N02AJ06, *N02AA59, N02AA79, N02AA08, N02AJ02, N02AJ03, N02AJ01, N02AA58, R05DA12,* R05DA04	CYP2D6 [a,b]
Desflurane	N01AB07	RYR1 [a], CACNA1S [a]
Desipramine	N06AA01	CYP2D6 [a], CYP2C19 [a]
Doxepin	N06AA12, *D04AX01*	CYP2D6 [a,b], CYP2C19 [a]
Efavirenz	J05AG03, J05AR06, *J05AR11*	CYP2B6 [a,b]
Eliglustat	A16AX10	CYP2D6 [b]
Enflurane	N01AB04	RYR1 [a], CACNA1S [a]
Escitalopram	N06AB10	CYP2C19 [a,b]
Oestrogens	G03AA, G03AB	F5/FvL [b]
Flecainide	C01BC04	CYP2D6 [b]
Flucloxacillin	J01CF05	HLA-B [b]
Fluorouracil	L01BC02, L01BC52	DPYD [a,b]
Fluvoxamine	N06AB08	CYP2D6 [a]
Haloperidol	N05AD01	CYP2D6 [b]
Halothane	N01AB01	RYR1 [a], CACNA1S [a]
Imipramine	N06AA02, N06AA03	CYP2C19 [a,b], CYP2D6 [a,b]
Irinotecan	L01XX19	UGT1A1 [b]
Isoflurane	N01AB06	RYR1 [a], CACNA1S [a]
Ivacaftor	R07AX02, R07AX30, R07AX31	CFTR [a]
Lamotrigine	N03AX09	HLA-B [b]
Lansoprazole	A02BC03, *A02BC06, A02BD07, A02BD10, A02BD03, A02BD09, A02BC53, A02BD02*	CYP2C19 [b]
Mercaptopurine	L01BB02	TPMT [a,b], NUDT15 [a,b]
Methoxyflurane	N02BG09	RYR1 [a], CACNA1S [a]
Metoprolol	C07AB02, *C07FX03, C07FB13, C07FB02, C07FX05, C07CB02, C07BB02, C07BB52*	CYP2D6 [b]
Nortriptyline	N06AA10	CYP2D6 [a,b], CYP2C19 [a]
Omeprazole	A02BC01, *A02BD05, A02BD01*	CYP2C19 [b]
Ondansetron	A04AA01	CYP2D6 [a]
Oxcarbazepine	N03AF02	HLA-B [a,b]
Pantoprazole	A02BC02, *A02BD04, A02BD11*	CYP2C19 [b]
Paroxetine	N06AB05	CYP2D6 [a,b]
Peginterferon alfa-2a	L03AB11, *L03AB61*	IFNL3 [a]
Peginterferon alfa-2b	L03AB10, *L03AB60*	IFNL3 [a]
Phenprocoumon	B01AA04	VKORC1 [b]
Phenytoin	N03AB02, *N03AB52*	CYP2C9 [a,b], HLA-B [a,b]
Pimozide	N05AG02	CYP2D6 [b]
Propafenone	C01BC03	CYP2D6 [b]
Rasburicase	V03AF07	G6PD [a]
Ribavirin	J05AP01	IFNL3 [a]
Risperidone	N05AX08	CYP2D6 [b]

Sertraline	N06AB06	CYP2C19 [a,b]
Sevoflurane	N01AB08	RYR1 [a], CACNA1S [a]
Simvastatin	C10AA01, *C10BX01, C10BA02, C10BA04, C10BX04*, A*10BH51*	SLCO1B1 [a,b]
Suxamethonium	M03AB01	RYR1 [a], CACNA1S [a]
Tacrolimus	L04AD02	CYP3A5 [a,b]
Tamoxifen	L02BA01	CYP2D6 [a,b]
Tegafur	*L01BC03*, L01BC53	DPYD [b]
Tioguanine	*L01BB03*	TPMT [a,b], NUDT15 [a,b]
Tramadol	N02AX02, *N02AJ14, N02AJ15, N02AJ13*	CYP2D6 [b]
Trimipramine	N06AA06	CYP2C19 [a], CYP2D6 [a]
Tropisetron	A04AA03	CYP2D6 [a]
Venlafaxine	*N06AX23*, N06AX16	CYP2D6 [b]
Voriconazole	J02AC03	CYP2C19 [a,b]
Warfarin	B01AA03	VKORC1 [a,b], CYP2C9 [a,b], CYP4F2 [a]
Zuclopenthixol	N05AF05	CYP2D6 [b]

[a] Actionable PGx guideline from CPIC
[b] Actionable PGx guideline from DPWG
The actionable PGx dosing guidelines provided by both CPIC and DPWG resulted in the presented 69 drugs (February 2020).[1,2] These PGx drugs were searched in Danish medical registries using their ATC codes. ATC codes in italic have never been or are currently not marketed in Denmark (June 2021).
Abbreviations: PGx: pharmacogenetics; CPIC: Clinical Pharmacogenetics Implementation Consortium; DPWG: Dutch Pharmacogenetics Working Group; ATC: Anatomical Therapeutic Chemical; DGI: drug-gene interaction.

Supplement Table 2. Prevalence of PGx prescription drug users in population cohort

Drug name		Birth cohort81 (1981-1994)[a]						Birth cohort95 (1995-2005)[a]					
		Overall		Sex				Overall		Sex			
		(N=15,894)		Female (N=7,817)		Male (N=8,077)		(N=14,081)		Female (N=6,866)		Male (N=7,215)	
		N	(%)	N	(%)	N	(%)	N	(%)	N	(%)	N	(%)
Allopurinol		15	(0.1)	5	(0.1)	10	(0.1)	<5	(0.0)	<5	(<0.1)	<5	(<0.1)
Amitriptyline	P	170	(1.1)	126	(1.6)	44	(0.5)	33	(0.2)	25	(0.4)	8	(0.1)
Aripiprazole	P	48	(0.3)	26	(0.3)	22	(0.3)	20	(0.1)	9	(0.1)	11	(0.2)
Atomoxetine	P	69	(0.4)	19	(0.2)	50	(0.6)	92	(0.7)	31	(0.5)	61	(0.8)
Atorvastatin		23	(0.1)	13	(0.2)	10	(0.1)	<5	(0.0)	<5	(<0.1)	<5	(<0.1)
Azathioprine		90	(0.6)	40	(0.5)	50	(0.6)	27	(0.2)	15	(0.2)	12	(0.2)
Carbamazepine	P	39	(0.2)	18	(0.2)	21	(0.3)	12	(0.1)	-	-	-	-
Citalopram	P	1160	(7.3)	792	(10.1)	368	(4.6)	48	(0.3)	35	(0.5)	13	(0.2)
Clopidogrel		15	(0.1)	5	(0.1)	10	(0.1)	<5	(0.0)	-	-	<5	(<0.1)
Codeine		1789	(11.3)	1188	(15.2)	601	(7.4)	374	(2.7)	230	(3.3)	144	(2.0)
Escitalopram	P	326	(2.1)	226	(2.9)	100	(1.2)	8	(0.1)	-	-	-	-
Oestrogens		-	-	6309	(80.7)	<5	(<0.1)	-	-	2338	(34.1)	<5	(<0.1)
Flucloxacillin		340	(2.1)	170	(2.2)	170	(2.1)	220	(1.6)	107	(1.6)	113	(1.6)
Haloperidol	P	7	(0.0)	<5	(<0.1)	<5	(<0.1)	-	-	-	-	-	-
Imipramine	P	41	(0.3)	24	(0.3)	17	(0.2)	17	(0.1)	5	(0.1)	12	(0.2)
Lamotrigine	P	214	(1.3)	140	(1.8)	74	(0.9)	70	(0.5)	33	(0.5)	37	(0.5)
Lansoprazole		1225	(7.7)	753	(9.6)	472	(5.8)	266	(1.9)	179	(2.6)	87	(1.2)
Metoprolol		216	(1.4)	165	(2.1)	51	(0.6)	46	(0.3)	28	(0.4)	18	(0.2)
Nortriptyline	P	56	(0.4)	41	(0.5)	15	(0.2)	9	(0.1)	-	-	-	-
Omeprazole		1111	(7.0)	722	(9.2)	389	(4.8)	440	(3.1)	269	(3.9)	171	(2.4)
Ondansetron		91	(0.6)	82	(1.0)	9	(0.1)	32	(0.2)	27	(0.4)	5	(0.1)
Oxcarbazepine	P	74	(0.5)	33	(0.4)	41	(0.5)	41	(0.3)	19	(0.3)	22	(0.3)
Pantoprazole		1185	(7.5)	708	(9.1)	477	(5.9)	331	(2.4)	229	(3.3)	102	(1.4)
Paroxetine	P	104	(0.7)	66	(0.8)	38	(0.5)	7	(0.0)	<5	(<0.1)	<5	(<0.1)
Pimozide	P	20	(0.1)	7	(0.1)	13	(0.2)	<5	(0.0)	<5	(<0.1)	<5	(<0.1)
Risperidone	P	109	(0.7)	53	(0.7)	56	(0.7)	41	(0.3)	12	(0.2)	29	(0.4)
Sertraline	P	753	(4.7)	482	(6.2)	271	(3.4)	149	(1.1)	98	(1.4)	51	(0.7)
Simvastatin		56	(0.4)	31	(0.4)	25	(0.3)	<5	(0.0)	-	-	<5	(<0.1)
Tramadol		1864	(11.7)	1035	(13.2)	829	(10.3)	237	(1.7)	137	(2.0)	100	(1.4)
Venlafaxine	P	344	(2.2)	235	(3.0)	109	(1.3)	26	(0.2)	-	-	-	-
Warfarin		51	(0.3)	40	(0.5)	11	(0.1)	8	(0.1)	-	-	-	-
Zuclopenthixol	P	13	(0.1)	-	-	-	-	<5	(0.0)	-	-	<5	(<0.1)

[a] *Birth cohort81 includes individuals born between 1981 and 1994, birth cohort95 includes individuals born between 1995 and 2005.*
The prevalence of PGx prescription drug users of 32 PGx drugs of the 45 identified PGx drugs is presented for the population cohort. For each birth cohort, the data is presented as a total and by sex. Prevalence below 5 is shown as <5. If the prevalence was below 5 in each column the drug was removed from this table (13 PGx drugs, i.e., clomipramine, doxepin, flecainide, fluorouracil, fluvoxamine, mercaptopurine, phenprocoumon, phenytoin, propafenone, tacrolimus, tamoxifen, trimipramine, voriconazole). Total numbers or by sex are omitted when counts of less than 5 could be deducted.
Abbreviations: P: psychotropic drug.

Supplement Table 3. Prevalence of PGx prescription drug users in combined mental disorder case cohorts

		Birth cohort81 (1981-1994)[a]						Birth cohort95 (1995-2005)[a]					
		Overall (N=37,437)		Sex				Overall (N=18,628)		Sex			
				Female (N=19,619)		Male (N=17,818)				Female (N=4,971)		Male (N=13,657)	
Drug name		N	(%)	N	(%)	N	(%)	N	(%)	N	(%)	N	(%)
Allopurinol		65	(0.2)	28	(0.1)	37	(0.2)	<5	(0.0)	<5	(<0.1)	<5	(0.0)
Amitriptyline	P	1289	(3.4)	917	(4.7)	372	(2.1)	68	(0.4)	38	(0.8)	30	(0.2)
Aripiprazole	P	4173	(11.1)	2308	(11.8)	1865	(10.5)	1070	(5.7)	387	(7.8)	683	(5.0)
Atomoxetine	P	3547	(9.5)	1415	(7.2)	2132	(12.0)	3165	(17.0)	797	(16.0)	2368	(17.3)
Atorvastatin		224	(0.6)	105	(0.5)	119	(0.7)	6	(0.0)	<5	(<0.1)	<5	(0.0)
Azathioprine		269	(0.7)	158	(0.8)	111	(0.6)	44	(0.2)	11	(0.2)	33	(0.2)
Carbamazepine	P	526	(1.4)	204	(1.0)	322	(1.8)	98	(0.5)	38	(0.8)	60	(0.4)
Citalopram	P	14696	(39.3)	9670	(49.3)	5026	(28.2)	564	(3.0)	327	(6.6)	237	(1.7)
Clomipramine	P	411	(1.1)	278	(1.4)	133	(0.7)	5	(0.0)	<5	(<0.1)	<5	(0.0)
Clopidogrel		57	(0.2)	42	(0.2)	15	(0.1)	<5	(0.0)	-	-	<5	(0.0)
Codeine		6850	(18.3)	4838	(24.7)	2012	(11.3)	681	(3.7)	271	(5.5)	410	(3.0)
Doxepin	P	28	(0.1)	12	(0.1)	16	(0.1)	-	-	-	-	-	-
Escitalopram	P	6204	(16.6)	4084	(20.8)	2120	(11.9)	157	(0.8)	93	(1.9)	64	(0.5)
Oestrogens		16522	(44.1)	16515	(84.2)	7	(0.0)	2392	(12.8)	2383	(47.9)	9	(0.1)
Flucloxacillin		1428	(3.8)	827	(4.2)	601	(3.4)	458	(2.5)	156	(3.1)	302	(2.2)
Fluvoxamine	P	42	(0.1)	35	(0.2)	7	(0.0)	5	(0.0)	<5	(<0.1)	<5	(0.0)
Haloperidol	P	235	(0.6)	126	(0.6)	109	(0.6)	20	(0.1)	9	(0.2)	11	(0.1)
Imipramine	P	244	(0.7)	140	(0.7)	104	(0.6)	22	(0.1)	8	(0.2)	14	(0.1)
Lamotrigine	P	4922	(13.1)	3362	(17.1)	1560	(8.8)	616	(3.3)	297	(6.0)	319	(2.3)
Lansoprazole		6009	(16.1)	3995	(20.4)	2014	(11.3)	573	(3.1)	305	(6.1)	268	(2.0)
Metoprolol		983	(2.6)	680	(3.5)	303	(1.7)	74	(0.4)	36	(0.7)	38	(0.3)
Nortriptyline	P	1348	(3.6)	923	(4.7)	425	(2.4)	24	(0.1)	12	(0.2)	12	(0.1)
Omeprazole		4985	(13.3)	3421	(17.4)	1564	(8.8)	823	(4.4)	373	(7.5)	450	(3.3)
Ondansetron		487	(1.3)	425	(2.2)	62	(0.3)	58	(0.3)	30	(0.6)	28	(0.2)
Oxcarbazepine	P	432	(1.2)	189	(1.0)	243	(1.4)	191	(1.0)	52	(1.0)	139	(1.0)
Pantoprazole		6070	(16.2)	3908	(19.9)	2162	(12.1)	717	(3.8)	389	(7.8)	328	(2.4)
Paroxetine	P	1699	(4.5)	1100	(5.6)	599	(3.4)	55	(0.3)	25	(0.5)	30	(0.2)
Phenytoin	P	20	(0.1)	11	(0.1)	9	(0.1)	7	(0.0)	<5	(<0.1)	<5	(0.0)
Pimozide	P	316	(0.8)	87	(0.4)	229	(1.3)	77	(0.4)	12	(0.2)	65	(0.5)
Risperidone	P	5332	(14.2)	2530	(12.9)	2802	(15.7)	1379	(7.4)	311	(6.3)	1068	(7.8)
Sertraline	P	11493	(30.7)	7504	(38.2)	3989	(22.4)	1855	(10.0)	955	(19.2)	900	(6.6)
Simvastatin		578	(1.5)	309	(1.6)	269	(1.5)	12	(0.1)	6	(0.1)	6	(0.0)
Tamoxifen		<10	(0.0)	-	-	-	-	-	-	-	-	-	-
Tramadol		8799	(23.5)	5644	(28.8)	3155	(17.7)	499	(2.7)	237	(4.8)	262	(1.9)
Venlafaxine	P	7925	(21.2)	5336	(27.2)	2589	(14.5)	184	(1.0)	117	(2.4)	67	(0.5)
Warfarin		197	(0.5)	140	(0.7)	57	(0.3)	18	(0.1)	5	(0.1)	13	(0.1)
Zuclopenthixol	P	821	(2.2)	430	(2.2)	391	(2.2)	18	(0.1)	12	(0.2)	6	(0.0)

[a] *Birth cohort81 includes individuals born between 1981 and 1994, birth cohort95 includes individuals born between 1995 and 2005.*
The prevalence of PGx prescription drug users of 37 of 45 identified PGx drugs is presented for the combined five mental disorder case cohorts. For each birth cohort, the data is presented as a total and by sex. Prevalence below 5 is shown as <5. If the prevalence was below 5 in each column the drug was removed from this table (8 PGx drugs i.e., flecainide, fluorouracil, mercaptopurine, phenprocoumon, propafenone, tacrolimus, trimipramine, voriconazole).
Abbreviations: SMD: severe mental disorder; P: psychotropic drug.

References:

1. KNMP (Royal Dutch Pharmacists Association). Pharmacogenetic Recommendations. last updated August 2020. Accessed February 20, 2020. https://www.knmp.nl/downloads/pharmacogenetic-recommendations-may-2020.pdf/view

2. Clinical Pharmacogenetics Implementation Consortium (CPIC). CPIC Guidelines. last updated August 2020. Accessed February 22, 2020. https://cpicpgx.org/guidelines/
